# Supplementary material for: Guiding Peptide Kinetics via Collective-Variable Tuning of Free-Energy Barriers
Source: J Chem Theory Comput. 2026 Apr 20;22(9):4573–80. doi: 10.1021/acs.jctc.6c00418 (PMC13173532; doi:10.1021/acs.jctc.6c00418)
Supplement: Supplementary file 1 [file ct6c00418_si_001.pdf]

Guiding Peptide Kinetics via Collective-Variable Tuning of Free-Energy Barriers

Alexander Zhilkin,[1] Muralika Medaparambath,[1,2] and Dan Mendels*[1,*]

[1] *The Wolfson Department of Chemical Engineering, Technion - Israel Institute of Technology, Haifa 32000, Israel*

[2] *Faculty of Mathematics, Technion - Israel Institute of Technology, Haifa 32000, Israel*

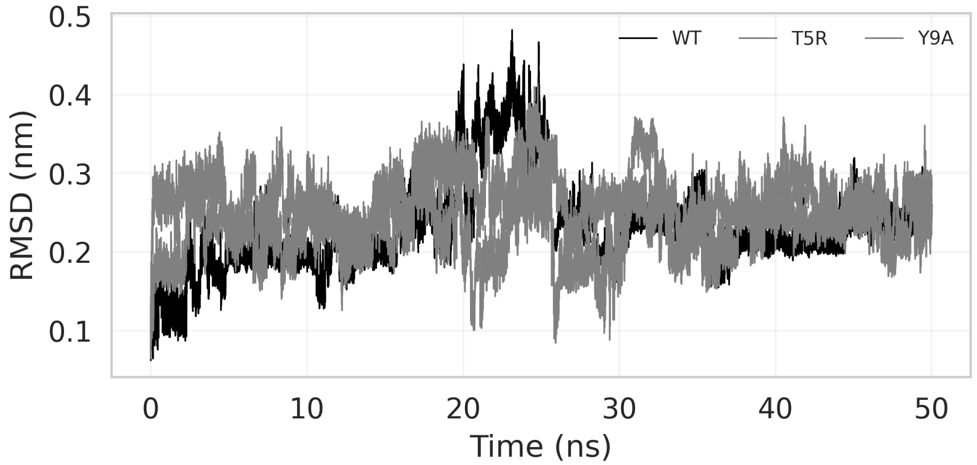

Figure S1: Chignolin WT and two point-mutations backbone RMSD from a reference folded structure corresponding to the enthalpic minimum of the native hairpin, computed along an unbiased short trajectory.

* danmendels@technion.ac.il

Table S1: Mutant-specific unfolding kinetics and HLDA separability for all simulated Chignolin variants at $t_{\mathrm{FPT}} = 0.34$ nm, with folded and unfolded state definitions given by $t_F = 0.25$ nm and $t_U = 0.57$ nm, respectively.

**Mutant**	**MFPT (μs)**	$\log(\mathrm{MFPT_{WT}}/\mathrm{MFPT_{mut}})$	**HLDA eigenvalue λ**
WT	0.625292	0.000000	4919.89
Y0A (Tyr→Ala)	0.404168	0.436389	7821.38
Y0E (Tyr→Glu)	0.309572	0.703029	8761.26
Y0Q (Tyr→Gln)	0.154364	1.398910	5988.56
Y0R (Tyr→Arg)	0.231474	0.993750	4223.07
D2A (Asp→Ala)	0.819860	-0.270915	6533.15
D2C (Asp→Cys)	8.187270	-2.572120	8717.71
D2E (Asp→Glu)	2.873120	-1.524940	7152.95
D2K (Asp→Lys)	0.285676	0.783361	7217.19
D2M (Asp→Met)	1.977810	-1.151530	4879.15
D2N (Asp→Asn)	0.395252	0.458695	6592.66
D2R (Asp→Arg)	0.130910	1.563710	5599.75
D2Y (Asp→Tyr)	0.673443	-0.074184	4939.27
P3C (Pro→Cys)	0.858174	-0.316588	5011.25
P3D (Pro→Asp)	0.0681503	2.216500	5290.57
P3M (Pro→Met)	2.623820	-1.434170	8467.28
P3R (Pro→Arg)	0.0600011	2.343860	4102.90
E4A (Glu→Ala)	0.164000	1.338350	5279.98
E4G (Glu→Gly)	1.221300	-0.669453	6107.72
E4R (Glu→Arg)	0.507556	0.208612	4883.88

Table S2: Table S1, continued.

**Mutant**	**MFPT (μs)**	$\log(\mathrm{MFPT_{WT}}/\mathrm{MFPT_{mut}})$	**HLDA eigenvalue λ**
E4Y (Glu→Tyr)	0.0610672	2.326240	5277.81
T5D (Thr→Asp)	0.284876	0.786166	4382.62
T5G (Thr→Gly)	1.213010	-0.662639	5007.47
T5R (Thr→Arg)	9.901650	-2.762240	6624.71
T5Y (Thr→Tyr)	0.0888015	1.951810	4515.27
T7D (Thr→Asp)	0.754653	-0.188039	7353.58
T7G (Thr→Gly)	0.0174231	3.580420	2080.98
T7Q (Thr→Gln)	1.115220	-0.578592	7664.95
T7R (Thr→Arg)	15.299200	-3.197340	8451.40
T7V (Thr→Val)	3.076060	-1.593190	6107.72
T7Y (Thr→Tyr)	0.290318	0.767243	5469.91
Y9A (Tyr→Ala)	0.0370596	2.825690	5926.13
Y9E (Tyr→Glu)	0.0444394	2.644090	2720.06
Y9G (Tyr→Gly)	0.0410230	2.724090	4726.74
Y9K (Tyr→Lys)	0.0165800	3.630020	4072.33
Y9Q (Tyr→Gln)	0.0360310	2.853840	3864.45
Y9R (Tyr→Arg)	0.3163610	0.681334	6439.89
Y9V (Tyr→Val)	0.0693590	2.198920	3872.36

Table S3: HLDA descriptor-wise contributions for Chignolin point mutations. Descriptors (e.g., d03) denote backbone distances between residues 0 and 3 (0-indexed). Blank entries indicate descriptors pruned during preprocessing due to Spearman correlation ($\rho > 0.93$).

descriptor<br>system	d03	d04	d05	d06	d07	d08	d09	d14	d15	d16	d17	d18	d19	d25	d26	d27	d28	d29	d36	d37	d38	d39	d47	d48	d49	d58	d59	d69
AYDPETGTWY	0.25	-0.42	0.38	-0.2	-	-0.011	-0.034	0.028	-	-	-0.036	-	-0.0055	-0.086	-0.063	0.086	0.063	-0.21	0.1	-0.06	-0.085	-0.082	-0.057	-0.051	0.53	0.074	-0.4	0.19
EYDPETGTWY	0.25	-0.4	0.33	-0.17	-0.032	-0.022	0.0029	0.11	-0.12	-0.053	-0.17	0.22	-0.098	-0.12	-0.017	0.23	-0.26	0.012	0.1	0.015	-0.14	-0.13	-0.18	0.1	0.37	0.13	-0.36	0.18
QYDPETGTWY	0.17	-0.36	0.34	-0.1	-0.049	-0.025	-0.0085	0.072	0.058	-0.037	-0.21	0.22	-0.083	-0.17	-0.023	0.32	-0.33	-0.012	0.068	0.015	-0.12	-0.053	-0.24	0.22	0.33	0.066	-0.32	0.11
RYDPETGTWY	0.098	-0.36	0.4	-0.078	-0.079	-0.023	-0.023	0.087	0.032	-0.083	-0.15	0.22	-0.11	-0.18	0.0073	0.3	-0.37	0.026	0.052	-0.023	-0.097	0.028	-0.22	0.23	0.29	0.064	-0.35	0.092
YYAPETGTWY	0.21	-0.42	0.42	-0.2	-	-	-0.056	-	-	-	-	-0.029	-	-0.12	0.0032	0.069	-	-0.21	0.051	-0.035	-0.085	0.0067	-0.11	0.047	0.52	0.086	-0.42	0.14
YYCPETGTWY	-	-0.11	-0.054	0.18	-	-	0.082	-	-	-	-	-	0.077	0.26	0.093	0.03	0.021	0.39	-0.074	0.17	0.37	-0.66	0.073	-0.27	0.17	-	-	-
YYDCETGTWY	0.14	0.16	-0.25	-0.2	0.088	0.12	0.041	-0.097	-0.15	0.22	0.15	-0.31	0.19	0.24	0.0019	-0.29	0.3	0.054	-0.042	0.027	0.21	-0.4	0.19	-0.26	-0.038	0.0059	0.18	0.066
YYDDETGTWY	0.19	-0.38	0.31	-0.084	-0.036	-0.061	0.0045	0.12	0.044	0.021	-0.26	0.22	-0.065	-0.2	-0.047	0.28	-0.24	-0.13	-	0.17	-0.23	0.0064	-0.3	0.3	0.27	-0.028	-0.19	0.098
YYDMETGTWY	0.17	-0.39	0.34	-0.078	-0.027	-0.049	-0.0033	0.11	0.026	-0.0063	-0.23	0.21	-0.048	-0.17	-0.096	0.29	-0.16	-0.17	0.089	0.082	-0.3	0.063	-0.28	0.29	0.27	-0.0076	-0.23	0.11
YYDPATGTWY	0.26	-0.49	0.32	-	-	-0.17	0.047	0.15	-0.031	-0.14	-0.1	0.25	-0.14	-0.056	-0.074	0.12	-0.044	-0.12	0.13	-0.0022	-0.22	-0.053	-0.084	0.017	0.43	0.055	-0.31	0.14
YYDPEDGTWY	0.31	-0.41	0.27	-0.15	-	-0.054	0.022	0.037	-	-	-0.049	-	-0.016	-0.058	-0.13	0.088	0.17	-0.29	0.13	-0.069	-0.12	-0.12	-0.024	-0.13	0.55	0.048	-0.3	0.18
YYDPEGGTWY	0.43	-0.34	0.2	-0.3	-	-	-	-0.0071	-	-	-	-	-	-0.075	-0.071	0.041	-	-0.23	0.14	-0.083	-0.071	-0.21	-0.039	-0.082	0.5	0.12	-0.31	0.25
YYDPERGTWY	0.32	-0.41	0.31	-0.23	-	-	-	0.029	-	-	-	-	-	-0.096	-0.021	-	-	-0.21	0.075	-0.041	-0.028	-0.16	-0.013	-0.12	0.55	0.12	-0.37	0.19
YYDPETGDWY	0.33	-0.37	0.33	-0.32	-	0.021	-0.035	0.033	-	-	-0.025	-	-0.029	-0.097	0.074	-0.04	-	-0.094	-	0.089	-0.059	-0.21	-0.031	-0.1	0.47	0.12	-0.39	0.26
YYDPETGGWY	-0.18	0.43	-0.32	-	-	0.16	-0.023	-0.083	-0.013	0.15	0.15	-0.31	0.23	0.044	-0.016	-0.14	0.29	-0.041	-0.063	0.0023	0.12	-0.0009	0.077	-0.12	-0.38	-0.048	0.39	-0.073
YYDPETGQWY	0.29	-0.48	0.37	-0.12	-0.061	-0.051	0.017	0.12	-0.078	0.0044	-0.094	0.12	-0.045	-0.049	-0.14	0.2	-0.068	-0.14	0.12	-0.0091	-0.17	-0.12	-0.14	0.11	0.42	0.023	-0.31	0.19
YYDPETGRWY	-0.34	0.41	-0.32	0.25	-	-	-	-	-	-	-	-	-	0.047	0.0057	-	-	0.12	-0.068	0.0029	0.021	0.24	-	0.078	-0.52	-0.089	0.38	-0.23
YYDPETGTWA	-	-	0.046	-	-	-0.089	-0.16	-	-	-	-0.2	-0.026	-	-0.22	-0.22	0.0013	-	-0.55	-0.09	0.1	-0.32	0.49	-0.035	0.0072	0.34	-	-0.2	-
YYDPETGTWE	0.23	-0.36	0.34	-0.084	-0.15	0.0055	0.028	-	-	-	-0.16	0.085	-0.058	-0.18	-0.12	0.36	-0.27	-0.075	0.051	-0.074	-0.0065	-0.14	-0.22	0.18	0.42	-0.011	-0.28	0.12
YYDPETGTWG	0.23	-0.32	0.29	-	-0.24	0.017	-	-	-	-	-0.1	-	-	-0.18	-0.16	0.4	-0.3	-0.083	0.051	-0.11	0.089	-0.22	-0.23	0.16	0.42	-0.022	-0.21	0.054
YYDPETGTWK	0.26	-0.34	0.16	-	-	-0.13	0.026	-0.0027	-	-	-0.076	-	-0.031	0.025	-0.36	0.12	0.23	-0.44	0.21	-0.15	-0.27	0.12	-0.0056	0.07	0.38	-0.099	-0.18	0.16
YYDPETGTWQ	-	0.036	-0.029	0.029	-	0.063	-0.013	-	-0.15	0.083	0.25	-0.3	0.21	0.12	-0.0058	-0.25	0.46	-0.15	-	-0.13	0.29	-0.2	0.26	-0.47	0.12	-	0.11	-0.046
YYDPETGTWR	-	-	0.15	-0.16	-0.43	0.036	0.0013	-	-0.065	0.01	0.11	-0.13	-0.24	-0.11	-0.2	0.022	0.0078	-0.34	-	-	-0.34	0.56	-	-	-	-	-0.16	0.22
YYDPETGTWV	-	0.2	-0.34	0.06	0.13	-0.024	0.088	-	-0.15	0.23	0.046	-0.22	0.2	0.16	-0.1	-0.16	0.42	-0.17	-0.054	0.061	0.11	-0.18	0.076	-0.18	-0.17	-0.11	0.48	-0.12
YYDPETGVWY	0.25	-0.15	0.1	-0.24	-	-	-	-	-	0.17	-0.18	-	-	-0.27	0.06	-	-	-0.51	-0.17	0.29	-0.31	0.31	-0.23	0.24	0.18	-	-	-
YYDPETGYWY	0.1	-0.016	0.078	-0.24	-	0.2	-0.037	0.014	-0.1	0.1	0.13	-0.22	0.23	0.053	0.13	-0.18	-	0.31	-0.066	0.064	0.32	-0.57	0.12	-0.29	0.12	0.15	-0.11	0.12
YYDPEYGTWY	0.26	-0.39	0.25	-	-	-0.17	-	-0.096	-	-	-	-	-	0.0016	-0.32	0.14	-	-0.31	0.14	-0.19	-0.084	0.02	-0.04	0.19	0.49	-0.17	-0.29	0.077
YYDPGTGTWY	0.25	-0.47	0.41	-0.14	-0.01	-0.092	0.0083	0.12	-0.056	-0.055	-0.11	0.19	-0.099	-0.065	-0.041	0.15	-0.11	-0.075	0.11	-0.025	-0.13	-0.082	-0.091	0.036	0.43	0.065	-0.37	0.17
YYDPRTGTWY	0.32	-0.43	0.36	-0.24	-	-0.027	-0.0047	0.047	-	-	-0.056	-	-0.023	-0.13	0.042	0.049	-	-0.17	-	0.035	-0.0019	-0.18	-0.074	-0.082	0.52	0.075	-0.34	0.18
YYDPYTGTWY	0.29	-0.062	-0.15	-	-	-	-	-0.17	-	-	-	-	-	-0.016	-0.34	0.2	-	-0.5	0.24	-0.2	-0.27	0.16	-0.23	0.17	0.38	0.12	-0.11	0.092
YYDRETGTWY	0.14	-0.38	0.32	-0.051	-	-0.077	-0.0086	0.14	0.031	-0.051	-0.24	0.27	-0.086	-0.15	-0.11	0.3	-0.14	-0.17	0.13	0.03	-0.33	0.13	-0.23	0.22	0.27	0.046	-0.26	0.11
YYEPETGTWY	0.34	-0.39	0.31	-0.25	-	-	-	-	-	-	-	-	-	-0.061	0.0096	-	-	-0.11	0.066	0.005	-0.017	-0.25	-0.021	-0.091	0.52	0.1	-0.38	0.23
YYKPETGTWY	0.26	-0.41	0.24	-	-	-0.16	0.071	0.11	-	-	-0.26	0.24	-0.057	-0.1	-0.17	0.34	-0.091	-0.2	0.13	0.036	-0.24	-0.078	-0.27	0.23	0.32	-0.077	-0.099	0.081
YYMPETGTWY	-	-	0.024	-	-	0.095	-0.0049	-	-	-	0.24	-0.27	0.19	0.056	0.12	-0.28	0.25	0.17	-0.069	-0.012	0.42	-0.44	0.27	-0.43	0.095	-	-	-
YYNPETGTWY	-	-	0.3	-	-0.48	0.17	0.0033	-	-0.13	0.031	0.13	-0.19	-0.0082	-0.22	-0.21	0.39	-0.33	0.0077	0.092	-0.37	0.27	-0.017	-0.06	-	0.023	-	-0.02	0.011
YYRPETGTWY	-	-	0.046	-	-	-0.089	-0.16	-	-	-	-0.2	-0.026	-	-0.22	-0.22	0.0013	-	-0.55	-0.09	0.1	-0.32	0.49	-0.035	0.0072	0.34	-	-0.2	-
YYYPETGTWY	0.24	-0.39	0.28	-	-	-0.17	-0.052	-0.061	-	-	-0.11	-	-0.076	-0.067	-0.18	0.23	-	-0.32	0.097	-0.16	0.0014	-0.03	-0.12	-0.0023	0.59	-0.048	-0.25	0.021

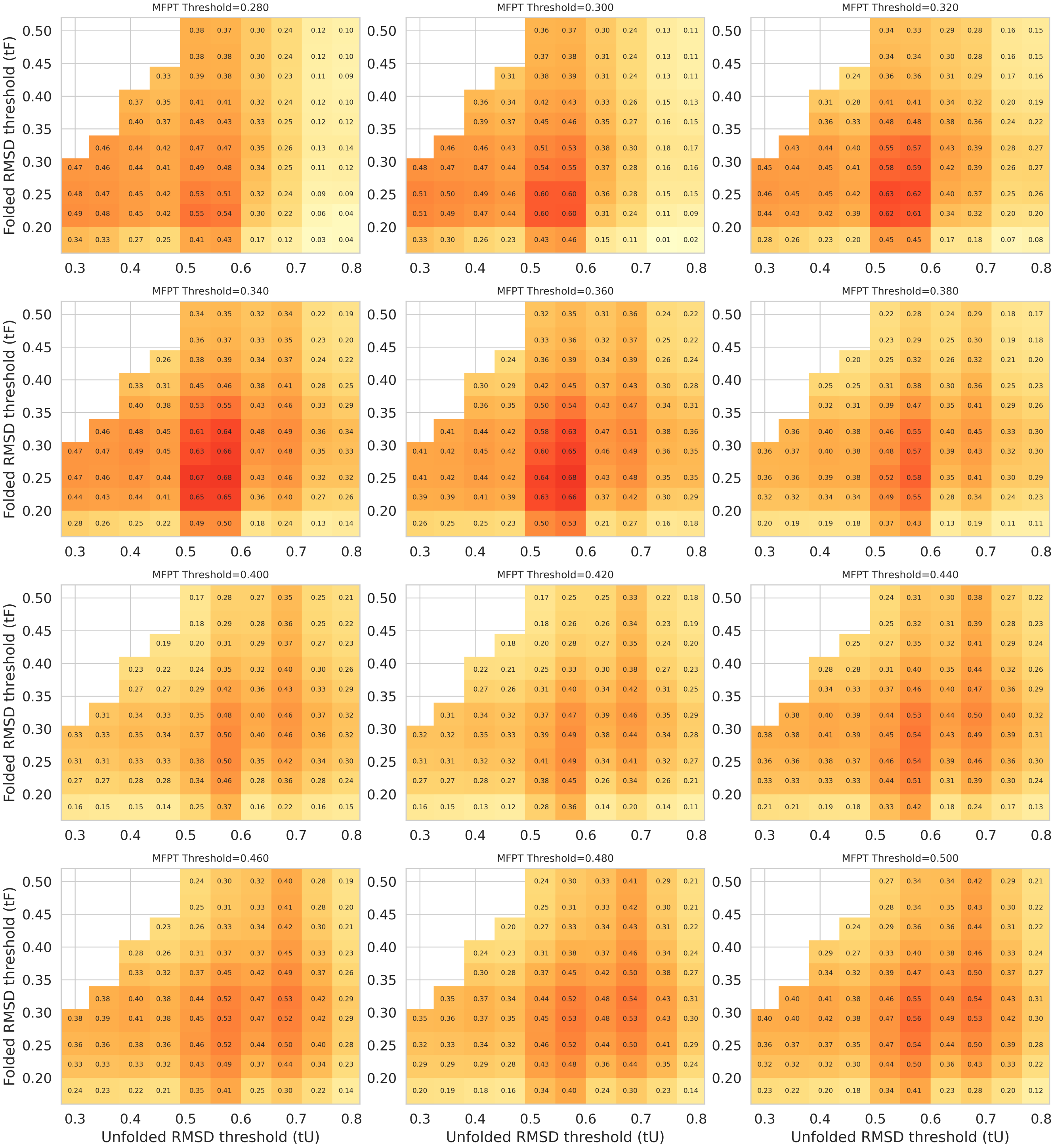

Figure S2: Grid of heatmaps showing the Pearson correlation coefficient r between the log MFPT ratio and the HLDA eigenvalue as a function of the RMSD thresholds used to define folded and unfolded states (t_F, t_U). Each panel corresponds to a different RMSD defining unfolding events (t_{FPT}), with color indicating correlation strength. Correlations persist over a range of values, demonstrating robustness of the HLDA–kinetics relationship to state definition.

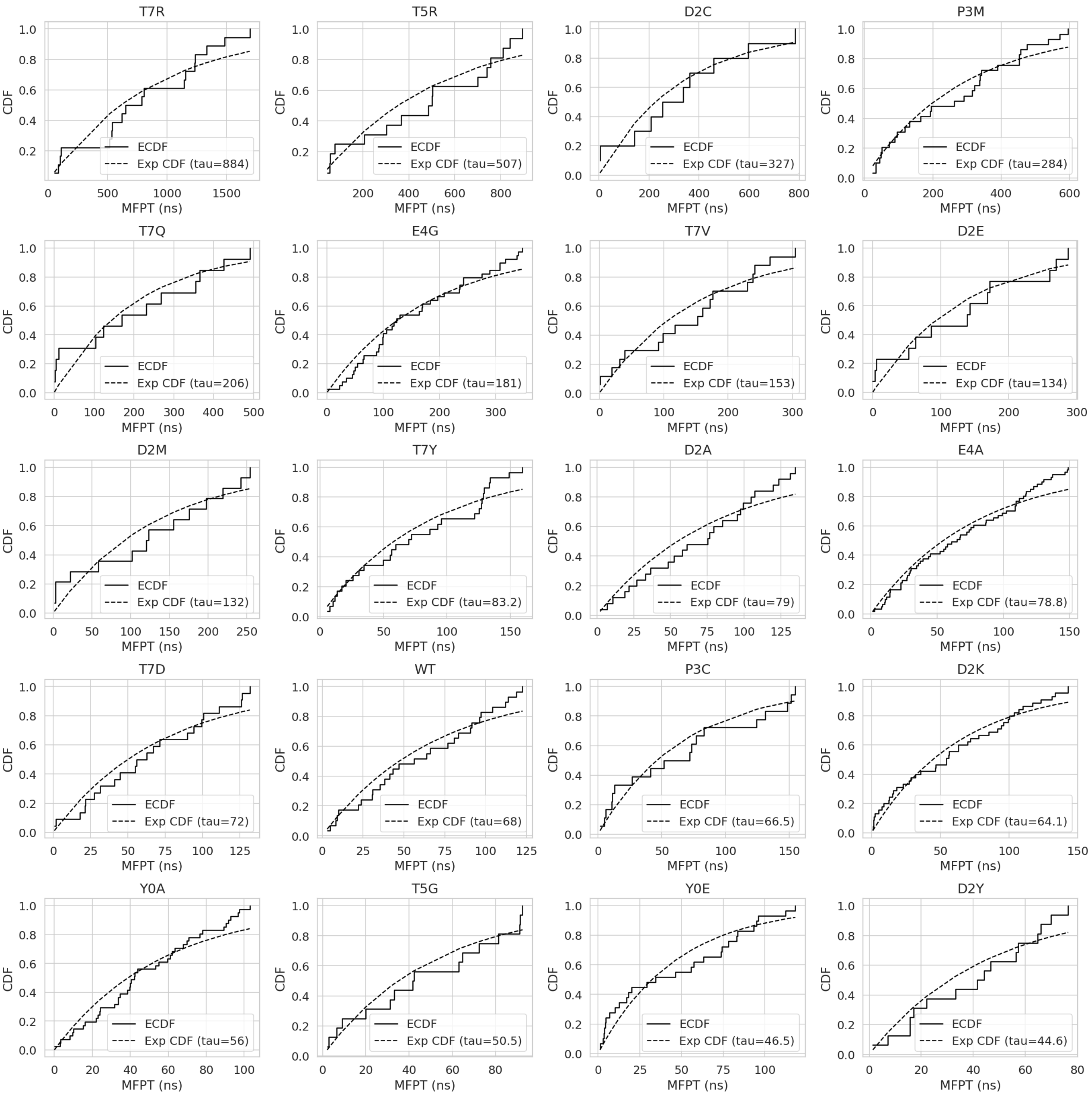

Figure S3: Empirical cumulative distribution function (ECDF) of first-passage times aggregated over all mutants, sorted from slowest to fastest unfolding, compared with the theoretical exponential CDF. The agreement supports an exponential first-passage-time model for the unfolding process.

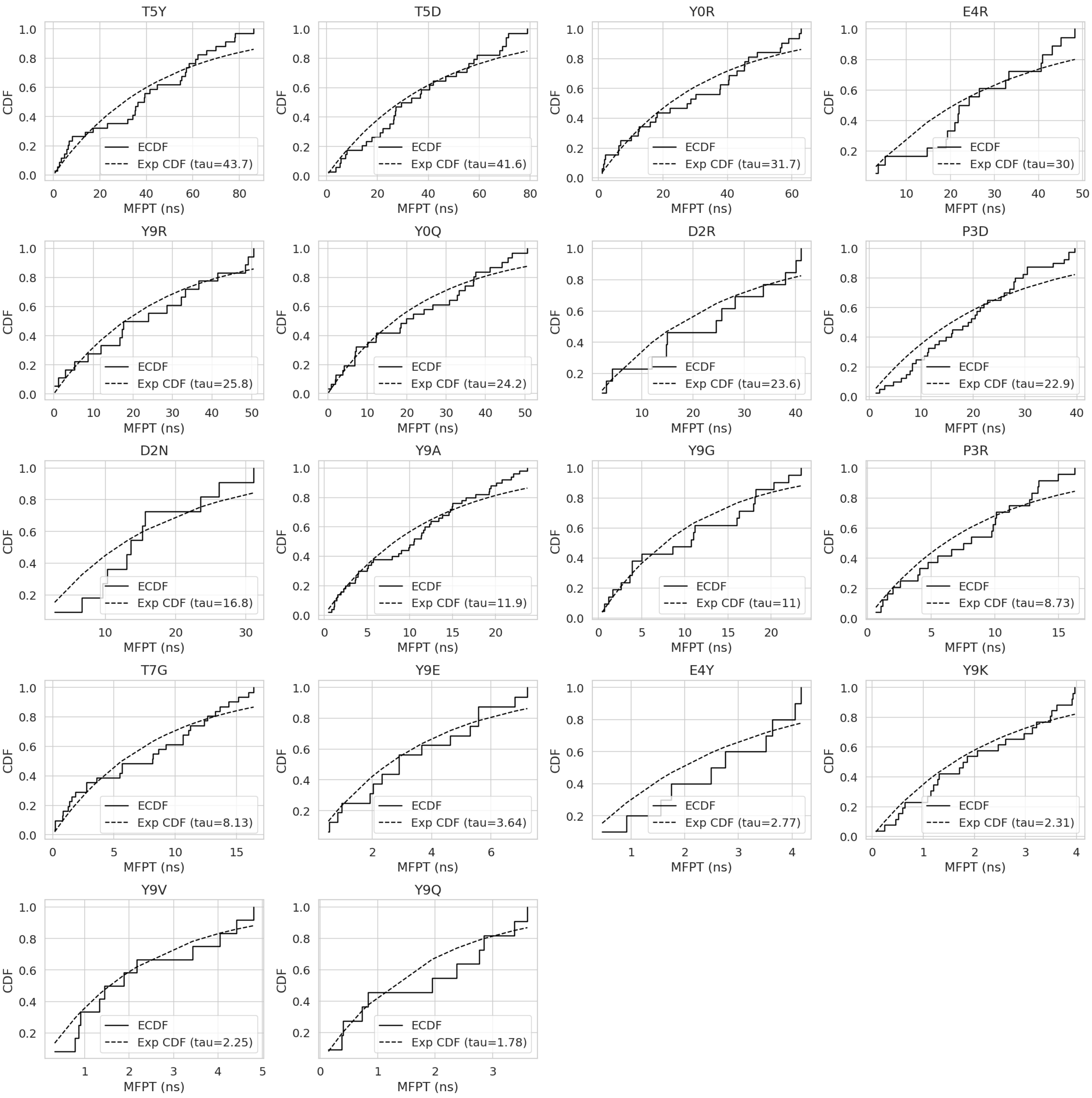

Figure S3: (continued)
